# Risk Factors for Soil-Transmitted Helminth Infections during the First 3 Years of Life in the Tropics; Findings from a Birth Cohort

**DOI:** 10.1371/journal.pntd.0002718

**Published:** 2014-02-27

**Authors:** Stefanie K. Menzies, Alejandro Rodriguez, Martha Chico, Carlos Sandoval, Nely Broncano, Irene Guadalupe, Philip J. Cooper

**Affiliations:** 1 Laboratorio de Investigaciones FEPIS, Quinindé, Esmeraldas Province, Ecuador; 2 Centro de Investigación en Enfermedades Infecciosas, Pontificia Universidad Católica del Ecuador, Quito, Ecuador; 3 Liverpool School of Tropical Medicine, Liverpool, United Kingdom; George Washington University, United States of America

## Abstract

**Background:**

Soil-transmitted helminths (STH) infect more than 2 billion humans worldwide, causing significant morbidity in children. There are few data on the epidemiology and risk factors for infection in pre-school children. To investigate risk factors for infection in early childhood, we analysed data prospectively collected in the ECUAVIDA birth cohort in Ecuador.

**Methods and Findings:**

Children were recruited at birth and followed up to 3 years of age with periodic collection of stool samples that were examined microscopically for STH parasites. Data on social, demographic, and environmental risk factors were collected from the mother at time of enrolment. Associations between exposures and detection of STH infections were analysed by multivariable logistic regression. Data were analysed from 1,697 children for whom a stool sample was obtained at 3 years. 42.3% had at least one STH infection in the first 3 years of life and the most common infections were caused by *A. lumbricoides* (33.2% of children) and *T. trichiura* (21.2%). Hookworm infection was detected in 0.9% of children. Risk of STH infection was associated with factors indicative of poverty in our study population such as Afro-Ecuadorian ethnicity and low maternal educational level. Maternal STH infections during pregnancy were strong risk factors for any childhood STH infection, infections with either *A. lumbricoides* or *T. trichiura*, and early age of first STH infection. Children of mothers with moderate to high infections intensities with *A. lumbricoides* were most at risk.

**Conclusions:**

Our data show high rates of infection with STH parasites during the first 3 years of life in an Ecuadorian birth cohort, an observation that was strongly associated with maternal STH infections during pregnancy. The targeted treatment of women of childbearing age, in particular before pregnancy, with anthelmintic drugs could offer a novel approach to the prevention of STH infections in pre-school children.

## Author Summary

Soil-transmitted helminths (STH) are intestinal worms that cause significant morbidity in school age and pre-school children in developing countries. Infections are associated with poverty, particularly through lack of access to sanitation and clean drinking water. Current control strategies rely on periodic anthelmintic treatment of schoolchildren but new strategies are required for STH control in young children. There are few data on modifiable risk factors in pre-school children. We investigated environmental and socioeconomic risk factors for STH infection in the first 3 years of life in a birth cohort from an STH-endemic region of Latin America. Our data provide evidence that maternal STH infections documented during pregnancy are an important risk factor for infection in young children, raising the possibility of a novel intervention for the prevention of STH-associated morbidity in early childhood through the deworming of women of childbearing age, in particular before pregnancy.

## Introduction

Soil-transmitted helminths (STH), including *A. lumbricoides lumbricoides*, *T. trichiura trichiura*, and hookworm, are estimated to infect more than 2 billion humans worldwide [1] of which 51 million children are considered to be at risk of morbidity [2]. An estimated 35 million or more disability-adjusted life years [3] have been attributed to STH infections.

Morbidity due to STH infections has primarily been associated with anaemia, malnutrition [4], stunting [5], and cognitive impairment [6]. Effects on childhood growth have been attributed to changes in appetite, digestion, nutrient absorption and iron loss [7].

Current strategies for the control of STH infections are primarily based upon periodic treatment of schoolchildren with anthelmintic drugs, and secondarily on education and improvements in sanitation. Treatment-based control strategies aim to control morbidity through reductions in the community transmission of STH infections [8].

Previous studies have shown that the main risk factors for STH infection are rural residency, low socioeconomic status and poor sanitation [2], [3]. The use of pit latrines and improved drinking water have been associated with a reduced prevalence of STH infections [4] while maternal STH infections with increased risk [9], indicating the importance of identifying risk factors amenable to targeted interventions.

There are few data on the epidemiology of STH infections and risk factors for infection in pre-school children. Such data are relevant to the control of STH infections because pre-school children constitute an important reservoir of infection and are at risk of morbidity. To investigate the epidemiology of STH infections in early childhood and to identify risk factors for infection, we analysed data collected prospectively during the first 3 years of life in the ECUAVIDA birth cohort in tropical Ecuador.

## Methods

### Study population and area

Details of the design and methodology for the ECUAVIDA birth cohort study, an investigator-driven study, are provided elsewhere [10]. Briefly, 2,404 newborns in the district of Quinindé in Esmeraldas Province, Ecuador, were recruited at the District hospital between November 2005 and December 2009. Inclusion criteria included being a healthy baby, the collection of a maternal stool sample, and planned residence in the District for at least 3 years. The study area is tropical at an elevation of up to 200 m, average annual temperature of 30°C and relative humidity of 75%, and with a population of ∼150,000 living in three towns and six rural parishes. The District is poor with limited access to clean water, sanitation, and basic services even within the towns.

### Study design and sample collection

All children recruited to the ECUAVIDA birth cohort study were eligible for inclusion in this analysis. Children were actively followed from birth to 3 years of age with collection of stool samples at 3, 7, 13, 18, 24, 30 and 36 months. Sample collection took place between November 2005 and December 2012 covering the period of cohort recruitment and the period to 3 years of age of the whole cohort. Stools at 3, 18, and 30 months were collected passively (mothers were asked to provide a sample at the next time point during the previous follow-up), while mothers were actively requested for stool samples at the respective ages for the remaining time points. Household members were also asked to provide one stool sample around the time that the mother was enrolled into the study. Data on risk factors and potential confounders were collected by a questionnaire that was administered to the child's mother by a trained member of the study team around the time of birth of the child. Categories included in the questionnaire were; maternal and paternal data (age, ethnicity, education, occupation, and number of live children [mother only]), urban versus rural location, socio-economic data (monthly income, number of material goods [household electrical appliances including refrigerator, television, Hi-Fi, and radio], a household electrical connection, household construction materials, sources of drinking water, and type of bathroom [for disposal of faeces]), number of sleeping rooms and number of people living in the household, and data on exposure to household pets, farming and farm animals. Household overcrowding was defined as number of people living in the household per bedroom. Data on number of anthelmintic treatments during the first 3 years of life was obtained from a questionnaire administered to the mother when the child was 7, 13, 24, and 36 months.

### Stool examinations

Single stool samples were collected and analysed for STH eggs and larvae by direct saline wet mounts (for detection of all STH eggs including *A. lumbricoides, T. trichiura, S. stercoralis*, hookworm, and tapeworms), Kato-Katz (for quantification of *A. lumbricoides* and *T. trichiura*) and formol-ether concentration (for detection of eggs/larvae of all STH and tapeworm parasites) methods [11]. *A. lumbricoides* and *T. trichiura* infection intensities were expressed as eggs per gram (epg) of faeces using the results of Kato-Katz. The intensities of hookworm and *S.stercoralis* were not evaluated because of low prevalence and intensities in children of this age. A positive sample was defined by the presence of at least one egg or larva from any of the three detection methods.

### Statistical analysis

A sample size of 1,697 individuals included in this analysis was estimated to provide over 80% power at P<0.05 to detect exposure effects on risk of any STH prevalence with effect sizes of or less than 0.56 (10% exposure prevalence), 0.68 (20%), 0.72 (30%, and 0.74 (40%). Any STH infection was defined by the presence of at least one STH ovum or larva in any stool sample. Potential risk factors evaluated included parental factors, child factors (gender, gestational age, and birth order), socioeconomic status and factors relating to the environment in which the child was living. A socio-economic status (SES) index was created using principal components analysis for categorical data by combining the socioeconomic variables. The first component that accounted for 30.0% of variation was divided into tertiles to represent low, middle, and high SES. The primary outcome was infection with any STH infection during the first 3 years of life. Secondary outcomes were age of first STH infection and any infection with *A. lumbricoides* or *T. trichiura* during the first 3 years of life. Estimates of effect for any STH infection and any infections with *A. lumbricoides* or *T. trichiura* during the first 3 years of life were calculated using multivariable logistic regression controlling for potential confounders using a backwards-stepwise procedure in which variables considered for inclusion were those with P<0.2 in univariate analyses. The variables, number of samples collected and number of anthelmintic treatments received and gender, were included in all analyses as potential confounding variables, the first because a greater number of samples collected increase the chances of detection of STH. Age of first STH infection was analysed by multinomial logistic regression, a technique that allows a single comparison group (uninfected) for more than one mutually exclusive outcome. This analysis allowed us to evaluate simultaneously, associations between risk factors and first STH infections acquired during the first, second and third years of life, respectively, compared to children without any documented STH infection. For multivariable multinomial logistic regression analysis, we used a backwards-stepwise method for selection of covariates – covariates considered in this analysis were those with P<0.005 in univariate analyses. The population-attributable fraction (PAFs) was calculated by: P_ew_ X (OR-1)/OR where P_ew_ is the prevalence of maternal STH infection among children with any STH infection during the first 3 years. Statistical significance was inferred by P<0.05. Analyses were done using SPSS (Version 16) and STATA version 10 (Statacorp, TX)

### Ethical approval and consent

The study protocol was approved by the ethics committee of the Hospital Pedro Vicente Maldonado, Universidad San Francisco de Quito, and Pontificia Universidad Catolica del Ecuador, Ecuador. The study is registered as an observational study (ISRCTN41239086). Informed written consent was obtained from the child's mother and from household members for the collection of stool samples. Children with positive stools for STH infections were treated with a single dose of 400 mg albendazole if aged 2 years or greater and with pyrantel pamoate (11 mg/kg) if aged less than 2 years, according to Ecuadorian Ministry of Public Health recommendations [12], [13].

## Results

### Selection of study participants for analysis

**Figure 1:**
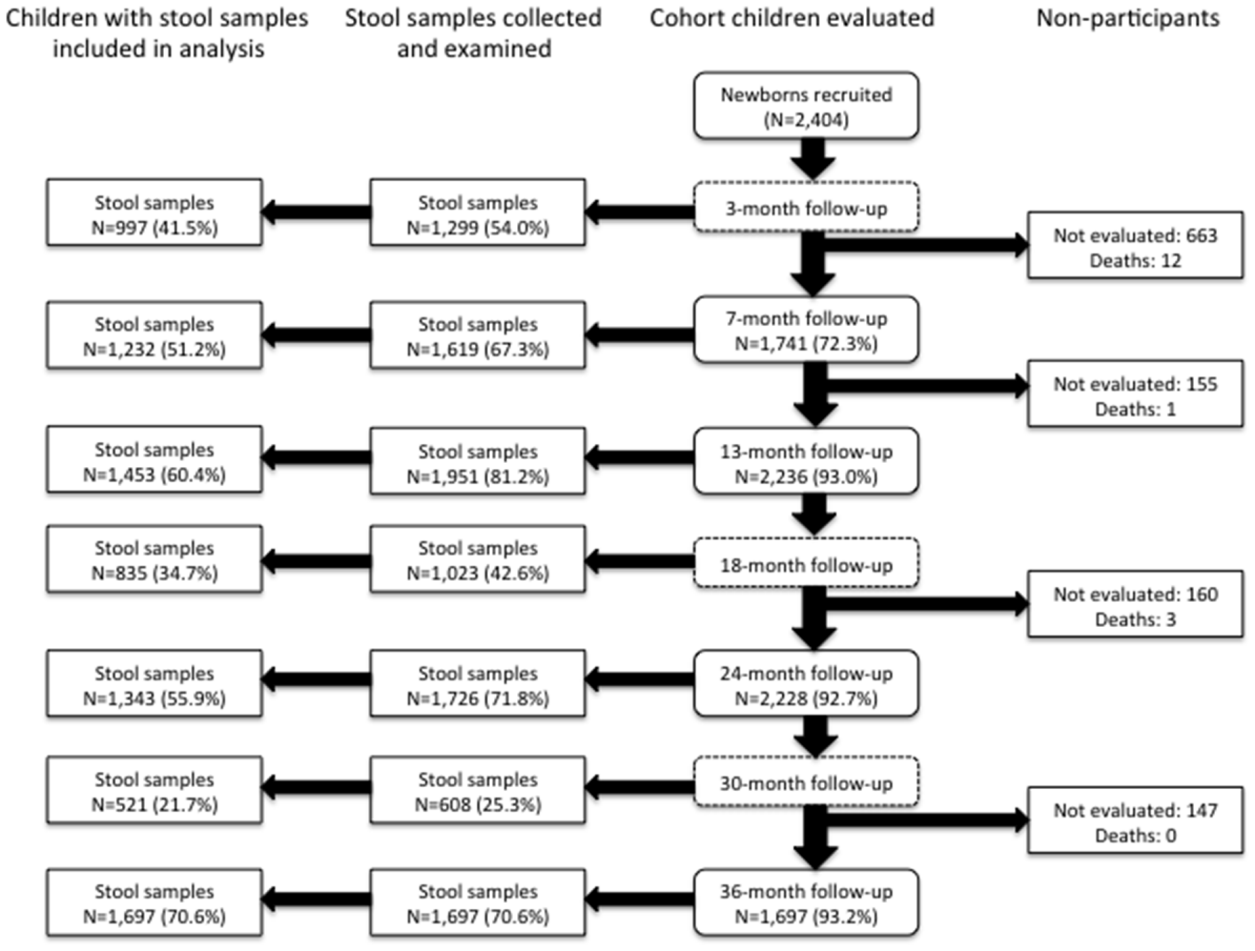
Flow diagram to show follow up of cohort to 3 years of age. The children included in the present analysis were those for whom a stool sample was collected at 3 years of age.

Of 2,404 newborns recruited, 1,697 (70.6%) of children had a stool sample examined at 3 years of age and these were the children included in the present analysis. Follow-up to 3 years of age for stool sampling for the whole cohort is illustrated in Figure 1. There were no significant differences between excluded and included children except for non-maternal STH infections among household members that were more frequent among excluded children (data provided in Table S1).

### Epidemiology of STH infections in pre-school children

**Figure 2:**
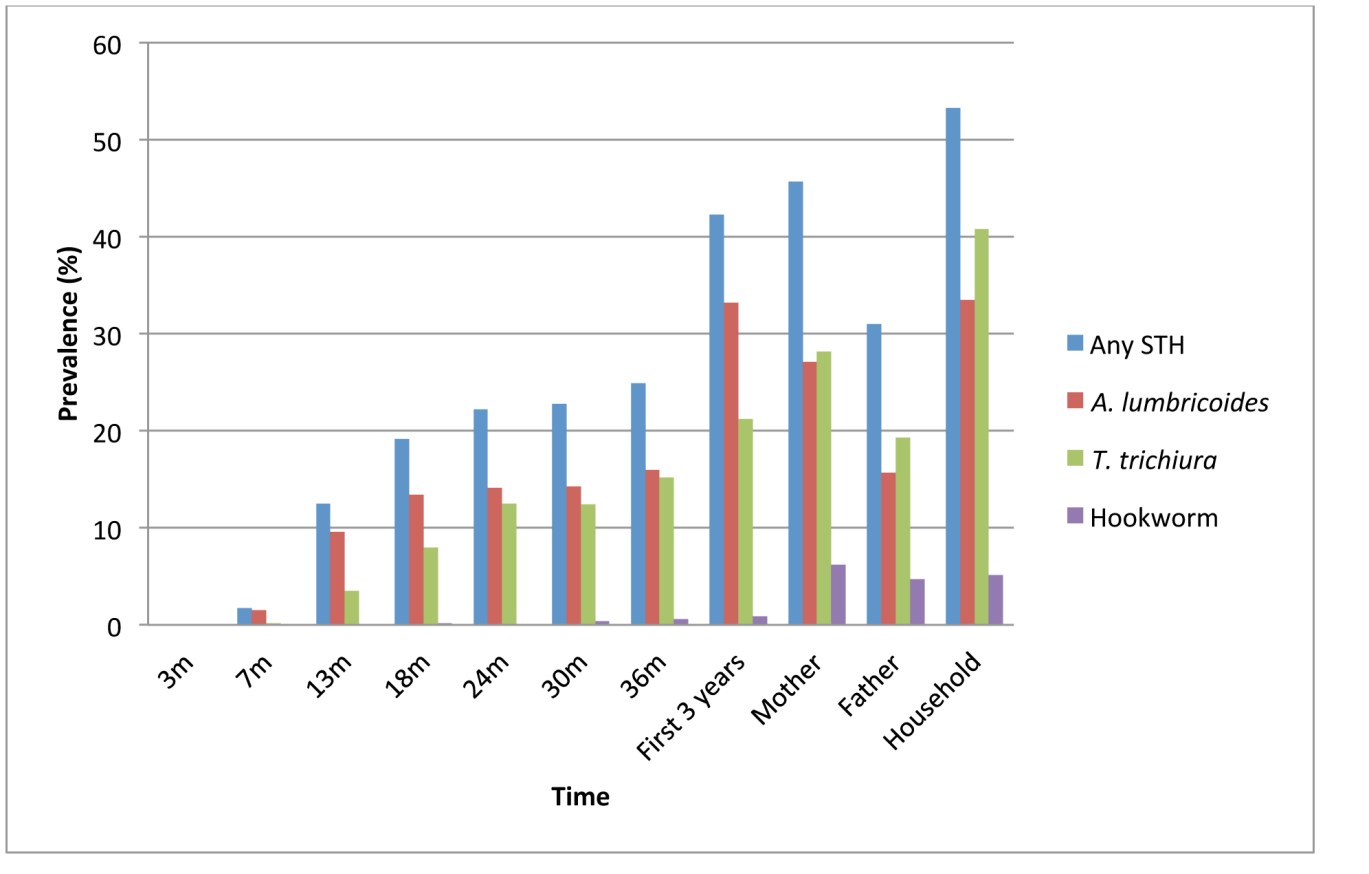
Prevalence of STH infections in children, parents, and other household members. Prevalence of STH infections in children is shown at regular age intervals during the first 3 years of life. The maternal stool sample was collected during the 3^rd^ trimester of pregnancy and from other household members soon after the child's birth.

**Figure 3:**
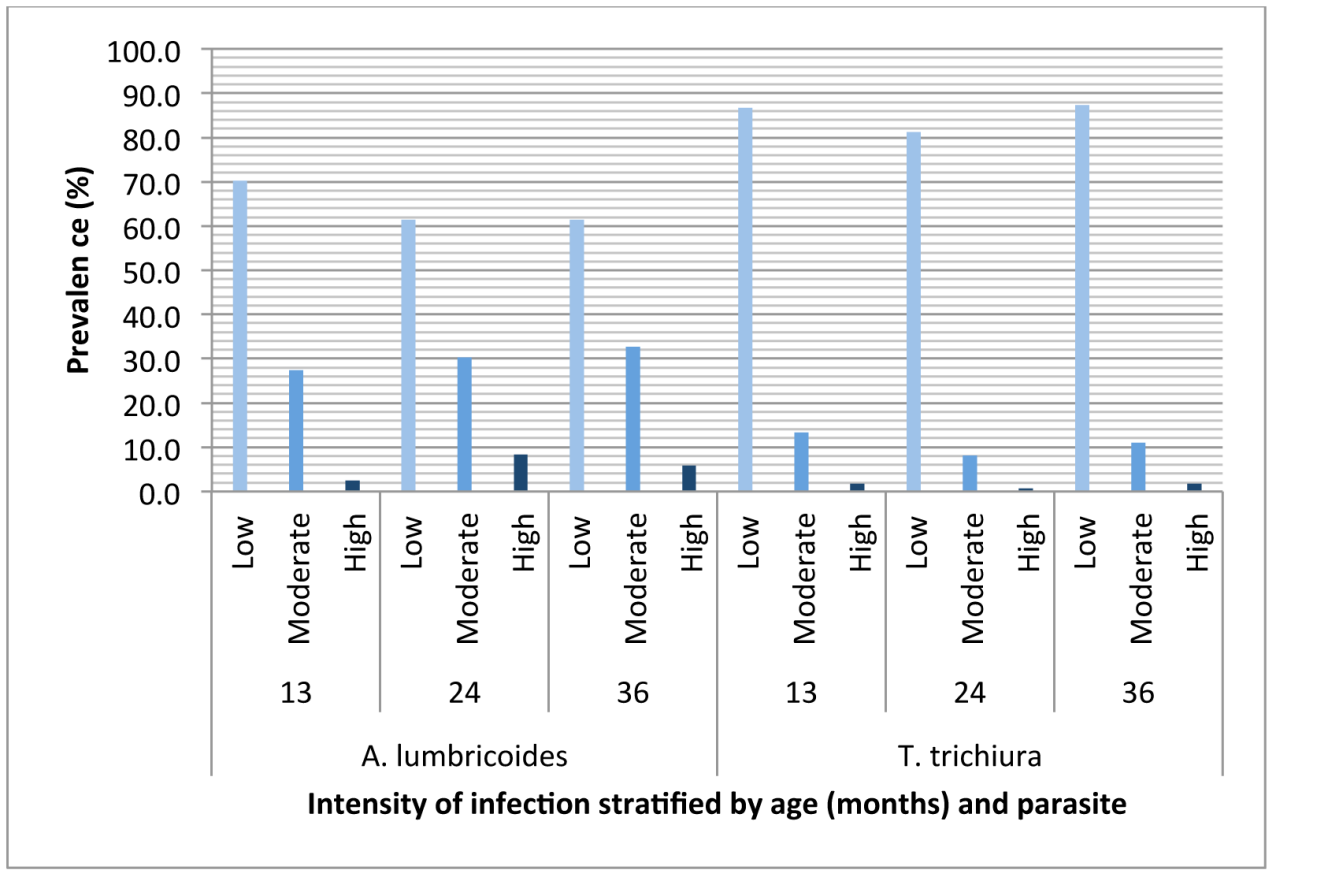
Cohort age-distributions of infection intensity categories with *A. lumbricoides* and *T. trichiura*. Infection intensities were estimated using the Kato-Katz method in eggs per gramme of stool and intensity groups were categorized using WHO guidelines [11]: *A. lumbricoides* (light- <5,000 epg; moderate = 5,000–49,999; heavy – ≥50,000); *T. trichiura* (light - <1,000 epg; moderate – 1,000–9,999; heavy – ≥10,000).

Of the 1,697 children analysed here, 718 (42.3%) were infected with at least one STH infection during the first 3 years of life. The most frequent STH infection was *A. lumbricoides* (33.2% of children had at least one documented infection during the first 3 years), followed by *T. trichiura* (21.2%), *Strongyloides stercoralis* (1.4%) and hookworm (0.9%). Other enteric parasites observed during the first 3 years of life included *Hymenolepis* spp. (2.2% of children), *Giardia lamblia* (44.8%) and *Entamoeba histolytica/dispar* (28.5%). The prevalence of STH infections by age is shown in Figure 2. STH infections appeared after 3 months of age and prevalence increased with age. The diagnostic methods used were not optimal for the detection of *S. stercoralis*: the infection was first detected at 13 months and prevalence did not vary substantially between 13 months and 3 years (0.4% at 13 months, 0.1% at 18 months, 0.6% at 24 months, 0.8% at 30 months, and 0.5% at 36 months. Mean age at first infection with an STH parasite among the study children was 23 months (SD 9 months, range 7–36 months). For comparisons with other studies, we stratified the children's infection intensities according to WHO recommendations [14]. The majority of infections were of low intensity (Figure 3). Geometric mean infection intensities among children with *A. lumbricoides* or *T. trichiura* infections, respectively, were: 13 months - 931 epg (range 35-182,910) or 244 epg (range 35-6,860); 24 months - 1,859 epg (range 35-176,750) or 248 epg (range 35-21,875); 36 months - 1,973 epg (range 35-283,930) or 243 epg (range 35-47,425). Anthelmintic treatments were widely available and were provided by the study team with a positive stool examination for STH infections and were also obtained directly from pharmacies by the mothers: 75.6% of children were treated at least once during the first 3 years of life and the proportions receiving anthelmintic treatments at 0–7, 8–13, 14–24, and 25–36 months were 0.8%, 16.5%, 44.5%, and 47.0%, respectively. No differences were observed in the number of anthelmintic treatments received between infected and uninfected children (Table 1). At enrolment, STH infections were present in 45.7% of mothers, 31.0% of fathers and 53.3% of other household members. The estimated prevalence of *S. stercoralis* among mothers was 4.0%.

**Table 1:** Characteristics of 1,697 study participants stratified by the presence or absence of any soil-transmitted helminth (STH) infection during the first 3 years of life.

Characteristic	Uninfected (n = 979) (N, %)		Infected (n = 718) (N, %)		P value
Child Factors					
Sex					
Male	497	50.8%	357	49.7%	0.671
Gestational age (weeks) [Mean/SD]	39	2	39	2	0.791
Birth order					
1–2	517	52.8%	306	42.6%	
3–4	311	31.8%	235	32.7%	
>5	151	15.4%	177	24.7%	<0.001
Maternal Factors					
Age [Mean/SD]	26	6	25	6	0.048
Ethnicity					
Afro-Ecuadorian	187	19.1%	259	36.1%	<0.001
Other	792	80.9%	459	63.9%	
Educational level					
Illiterate	103	10.5%	155	21.6%	
Complete primary	562	57.4%	448	62.4%	
Complete secondary	314	32.1%	115	16.0%	<0.001
Paternal Factors					
Age [Mean/SD]	30	8	30	9	0.516
Ethnicity					
Afro-Ecuadorian	175	18.3%	204	29.4%	<0.001
Other	779	81.6%	491	70.6%	
Educational level					
Illiterate	114	12.4%	133	20.9%	
Complete primary	486	52.8%	354	55.7%	
Complete secondary	321	34.9%	149	23.4%	<0.001
Socioeconomic status					
Low	328	33.5%	312	43.5%	
Medium	303	30.9%	230	32%	
High	348	35.5%	176	24.5%	<0.001
Environmental Factors					
Area of residence					
Urban	652	66.6%	528	73.5%	0.002
Household overcrowding					
≥3 people	511	65.0%	490	80.7%	<0.001
Maternal STH Infections					
Any STH infection					
Yes	346	35.5%	425	59.5%	<0.001
*A. lumbricoides* infection					
Yes	184	18.9%	273	38.2%	<0.001
*A. lumbricoides* intensity (epg)					
Negative	790	81.1%	441	61.7%	
Light	174	17.9%	200	28.0%	
Moderate	10	1.0%	67	9.4	
Heavy	0	0%	6	0.8%	<0.001
*T. trichiura* infection					
Yes	197	20.2%	279	39.1%	<0.001
*T. trichiura* intensity (epg)					
Negative	777	79.8%	438	61.2%	
Light	180	18.5%	224	31.4%	
Moderate	17	1.7%	47	6.6%	
Heavy	0	0%	6	0.8%	<0.001
Hookworm					
Yes	41	4.2%	63	8.8%	<0.001
Paternal STH infection					
Yes	82	23.9%	104	40.9%	<0.001
Other household member with STH					
Yes	408	41.7%	428	59.6%	<0.001
Number of stool samples from child					
1–4	442	45.1%	288	40.1%	
≥5	537	54.9%	430	59.9%	0.022
Number of anthelmintic treatments*					
Received by child					
0	250	25.5%	164	22.8%	
1	451	46.1%	323	45%	
≥2	278	28.4%	231	32.2%	0.191

P values were calculated using Chi-squared or Student's t tests, as appropriate. Ethnicity ‘other’ represents: mothers; 1,245 Mestizo/6 Indigenous; fathers 1264 Mestizo/6 Indigenous. Socioeconomic status represents tertiles of z scores obtained using a factor analysis. Overcrowding is defined as the number of people living in the household per sleeping room. STH infections were detected using direct saline, Kato-Katz and formol-ether concentration methods. SD – standard deviation. Infection intensities were estimated using the Kato-Katz method. STH infection intensity categories were: *A. lumbricoides* (light- <5,000 eggs per gramme of stool [epg];; moderate = 5,000–49,999; heavy – ≥50,000); *T. trichiura* (light - <1,000 epg; moderate – 1,000–9,999; heavy – ≥10,000).

*Treatments with any of: albendazole, mebendazole, oxantel/pyrantel, piperazine, nitazoxanide, and flubendazole. Numbers of missing values (brackets) were: gestational age (312), maternal ethnicity (6), paternal ethnicity (54), paternal educational level (140), household overcrowding (304), maternal STH infection (9), and paternal STH infection (1100).

### Risk factors associated with any STH infection

The distributions of covariates between children with any documented STH infection and those without a documented infection are shown in Table 1. Factors that were significantly more frequent among infected children were; being lower in the birth order, having a younger mother or Afro-Ecuadorian mother or illiterate mother, being of lower socioeconomic status, urban residence, living in a more crowded household, having a mother infected with an STH parasite during pregnancy particularly having a mother with moderate to high parasite burdens with *A. lumbricoides* or *T. trichiura*, and having a father or other household member infected with an STH parasite after the child's birth. The risk of infection was greater among children who provided 5 or more stool samples during the 3 years of observation. The number of anthelmintic treatments received by the child did not affect the risk of having any STH infection. There was some evidence that the prevalence of *S. stercoralis* was higher among mothers of children with STH infections compared to those without (6.0% vs. 2.3%, P<0.001). The results of univariate and multivariable analyses are shown in Table 2. In multivariable analyses, being lower in the birth order, maternal Afro-Ecuadorian ethnicity and younger age, being of low socioeconomic status, urban residence, and household overcrowding were significant independent predictors of STH infection during the first 3 years of life. Intensity of maternal *A. lumbricoides* infection produced the highest odds ratios; children were 11.6 times more likely to have STH if their mothers had moderate to heavy infection intensities with *A. lumbricoides*. This estimate was imprecise with high confidence intervals but is consistent with an analysis in which maternal *A. lumbricoides* infection intensities were stratified as tertiles and the highest tertile was compared with negatives (adj. OR 4.46 (95% CI 2.68–7.41), O<0.001) (data not shown). Having household members with STH infections also increased the child's chance of having an STH infection as did the number of stool samples collected for each child. In a separate analysis, we estimated the association between any maternal STH infection and any STH infection in children, excluding the other maternal STH variables shown in Table 2, giving an adjusted OR of 1.88 (95% CI 1.46–2.41, P<0.001). This estimate corresponds to a fraction of child STH infections attributable to maternal STH infections (PAF%) of 27.9%. To distinguish the effects of increased risk of child infection associated with STH during pregnancy from that of having a mother with STH infection during the first year of life, we stratified the data according to whether the mother was the child's primary carer during the first year of life or not: data were available for 1,639 children of whom mothers were the primary carer for 95.1%. The association between maternal STH infection and any child infections was greater among children with primary mother carers (adj. OR 1.84, 95% CI 1.40–2.41, P<0.001) compared to those with non-mother carers (adj. OR 0.35, 95% CI 0.05–2.58, P = 0.305) although the latter group consisted of only 81 children (data not shown).

**Table 2:** Univariate and multivariable associations between risk factors and having any STH infection during the first 3 years of life.

Variable	Univariate		Multivariable	
	OR (95% CI)	P value	OR (95% CI)	P value
Child Factors				
Sex: Male vs. Female	0.96 (0.79–1.16)	0.671		
Gestational age: <39 vs. ≥39 weeks	0.95 (0.76–1.19)	0.639		
Birth Order: ≥5^th^ vs. <5^thh^	1.79 (1.41–2.29)	<0.001	1.85 (1.31–2.60)	0.001
Maternal Factors				
Age: <26 vs. ≥26 years	1.19 (0.98–1.44)	0.082	1.52 (1.15–2.01)	0.003
Ethnicity: Afro vs. Other	2.38 (1.91–2.97)	<0.001	2.11 (1.61–2.75)	<0.001
Educational level				
Primary vs. Illiterate	0.53 (0.40–0.70)	<0.001		
Secondary vs. Illiterate	0.24 (0.18–0.34)	<0.001		
Paternal Factors				
Age: <30 vs. ≥30 years	0.88 (0.73–1.07)	0.197		
Ethnicity: Afro vs. Other	1.84 (1.46–2.31)	<0.002		
Educational level				
Primary vs. Illiterate	0.62 (0.47–0.83)	0.001		
Secondary vs. Illiterate	0.40 (0.29–0.55)	<0.001		
Socioeconomic status				
Medium vs. Low	0.80 (0.63–1.01)	0.056	0.79 (0.60–1.06)	0.118
High vs. Low	0.53 (0.42–0.68)	<0.001	0.54 (0.40–0.74)	<0.001
Environmental Factors				
Area of residence: Urban vs. Rural	1.39 (1.13–1.72)	0.002	**1.72 (1.31–2.27)**	<0.001
Household overcrowding: ≥3 vs. <3	2.25 (1.76–2.89)	<0.001	**1.81 (1.38–2.39)**	<0.001
Maternal STH Infections				
Any geohelminth: Yes vs. No	2.67 (2.19–3.26)	<0.001		
*A. lumbricoides*: Yes vs. No	2.66 (2.13–3.31)	<0.001		
*A. lumbricoides* intensity (epg)				
Light vs. Negative	2.07 (1.63–2.62)	<0.001	1.50 (1.13–1.99)	0.005
Moderate/heavy vs. Negative	13.1 (6.7–25.6)	<0.001	**11.6 (4.83–27.8)**	**<0.001**
*T. trichiura:* Yes vs. No	2.53 (2.04–3.14)	<0.001		
*T. trichiura* intensity (epg)				
Light vs. Negative	2.21 (1.76–2.77)	<0.001		
Moderate/heavy vs. Negative	5.53 (3.16–9.67)	<0.001		
Hookworm: Yes vs. No	2.20 (1.47–3.30)	<0.001		
Paternal STH infection: Yes vs. No	1.83 (1.35–2.49)	<0.001		
Household member with STH infection: Yes vs. No	2.07 (1.70–2.51)	<0.001	**1.44 (1.13–1.83)**	**0.003**
Number of stool samples from child				
≥5 vs. 1–4	1.23 (91.01–1.49)	0.039	**1.28 (1.01–1.62)**	**0.041**
Number of anthelmintic treatments				
1 vs. 0	1.09 (0.86–1.39)	0.48		
≥2 vs. 0	1.27 (0.97–1.65)	0.078		

Multivariable analyses included data from 1,381 children for whom complete data were available. Paternal STH infection was excluded from the multivariate model because of missing data. Odds ratios (ORs), 95% confidence intervals (95% CI) were estimated using logistic regression. STH were detected using all 3 microscopic detection methods. Overcrowding was defined as number of household members per sleeping room. SES (socioeconomic) index shows tertiles of Z scores calculated using principal components analysis. Paternal and maternal age, overcrowding, and gestational age used the mean as cut-off. STH infection intensity categories were: *A. lumbricoides* (light- <5,000 eggs per gramme of stool [epg]; moderate = 5,000–49,999; heavy – ≥50,000); *T. trichiura* (light - <1,000 epg; moderate – 1,000–9,999; heavy – ≥10,000).

### Risk factors associated with *A. lumbricoides* or *T. trichiura* infections

Most STH infections during the first 3 years of life were caused by *A. lumbricoides*. Risk factors for any *A. lumbricoides* or any *T. trichiura* infection during the first 3 years of life in multivariable analyses were similar to those documented for any STH infection and showed strong associations with maternal STH infections: 1) Any *A. lumbricoides* infections was associated with greater maternal infection intensities with *A. lumbricoides* (Moderate/heavy vs. uninfected, adj. OR 3.88, 95% CI 2.12–7.08, P<0.001) and maternal *T. trichiura* infections (adj. OR 1.38, 95% CI 1.05–1.82, P = 0.021); 2) Any *T. trichiur*a infection was associated with having a mother with greater maternal infection intensities with *A. lumbricoides* (Moderate/high vs. uninfected, adj. OR 5.85, 95% CI 3.29–10.40, P<0.001) or infection with *T. trichiura* (adj. OR 1.71, 95% CI 1.36–2.56, P<0.001) during pregnancy. (Complete results are provided in the Table S2).

### Risk factors associated for age at first infection with any STH parasite

Multivariable analyses showed that maternal infections with either *A. lumbricoides* or *T. trichiura* were consistently strong predictors of age at first infection with STH parasites across the first 3 years of life (Table 3). Maternal infection with *A. lumbricoides* during pregnancy doubled the odds of a child being infected during the first year of life (adj. OR 2.34, 95% CI 1.61–3.40) - a significant effect was seen also for first infections acquired in the second year of life. Similar effects were observed for age of first infection among children born to mothers with *T. trichiura* infections. Maternal Afro-Ecuadorian ethnicity and low educational level were also associated with an increased risk across the first 3 years of life, although the association was not significant for low maternal educational level in the second year of life. Being lower in the birth order and having a household member with an STH infection were associated only with early infections with STH in children (i.e. first year of life). Household overcrowding was associated with first infections after the 1^st^ year of life.

**Table 3:** Univariate and multivariable polytomous logistic regressions for factors associated with age of first infection with any STH parasite.

Variable	Univariate			Multivariable		
	First year*	Second year	Third year	First year	Second year	Third year
	OR (95% CI) P value	OR (95% CI) P value	OR (95% CI) P value	OR (95% CI) P value	OR (95% CI) P value	OR (95% CI) P value
Birth Order ≥5 vs. <5	3.0 (2.13–4.20) <0.001	1.59 (1.15–2.19) 0.005	1.29 (0.88–1.90) 0.196	2.04(1.36–3.07) 0.001	1.24(0.85–1.80) 0.261	0.87(0.55–1.36) 0.537
Maternal Ethnicity Afro-Ecuadorian vs. Other	2.31 (1.66–3.21) <0.001	2.14 (1.61–2.83) <0.001	2.06 (1.49–2.86) <0.001	1.82(1.25–2.65) 0.002	1.92(1.40–2.63) <0.001	1.92(1.34–2.75) <0.001
Maternal Educational Level ≥Primary vs. Illiterate	0.31(0.214–0.449) <0.001	0.53 (0.37–0.75) <0.001	0.50 (0.34–0.74) 0.001	0.52(0.33–0.81) 0.004	0.73(0.48–1.11) 0.138	0.60(0.38–0.96) 0.031
Paternal Ethnicity Afro-Ecuadorian vs. Other	1.71 (1.20–2.44) 0.003	1.83 (1.36–2.47) <0.001	1.74 (1.23–2.39) 0.001			
Paternal Educational level ≥Primary vs. Illiterate	0.43 (0.29–0.63) <0.001	0.55 (0.39–0.79) <0.001	0.70(0.46–1.08) 0.106			
SES Index Medium/High vs. Low	0.54(0.40–0.74) <0.001	0.70(0.53–0.91) 0.008	0.64(0.47–0.87) 0.004			
Area of residence Urban vs. Rural	1.16(0.83–1.62) 0.385	1.33(1.0–1.78) 0.520	1.43(1.02–2.02) 0.038			
Household overcrowding ≥3 vs. <3	2.02 (1.46–2.80) <0.001	2.05 (1.55–2.70) <0.001	1.93 (1.40–2.64) <0.001	1.21(0.84–1.76) 0.303	1.82(1.34–2.48) <0.001	1.82(1.28–2.58) <0.001
Maternal STH Infection Yes vs. No	4.58 (3.26–6.43) <0.001	2.25 (1.72–2.93) <0.001	1.93 (1.43–2.61) <0.001			
*A. lumbricoides* Yes vs. No	4.10(2.97–5.66) <0.001	2.34(1.76–3.11) <0.001	1.90(1.36–2.66) <0.001	2.34(1.61–3.40) <0.001	1.74(1.25–2.42) 0.001	1.39(0.95–2.04) 0.090
T. trichiura Yes vs. No	3.73(2.71–5.14) <0.001	2.07(1.56–2.75) <0.001	1.98(1.43–2.74) <0.001	2.57(1.77–3.73) <0.001	1.51(1.08–2.11) 0.015	1.46(1.0–2.13) 0.049
Hookworm Yes vs. No	2.67(1.55–4.62) <0.001	1.88(1.12–3.18) <0.001	2.02(1.13–3.61) 0.019			
Yes vs. No	<0.001	<0.001	0.019			
Household member with STH infection Yes vs. No	2.71(1.97–3.74) <0.001	1.87(1.44–2.44) <0.001	1.76(1.30–2.38) <0.001	1.59(1.08–2.33) 0.019	1.25(0.92–1.70) 0.162	1.26(0.89–1.79) 0.193

Multivariable analyses included data from 1,381 children for whom we had complete data. Associations between risk factors and age at first infection were compared to children without any infection in the first 3 years of life using univariate and multivariable multinomial logistic regression. STH were detected using all 3 microscopic detection methods. Variables with more than 2 groups in Tables 1 and 2 were redefined as binary. Overcrowding was defined as number of household members per sleeping room using the mean as cut-off. SES (socioeconomic) index shows tertiles of Z scores calculated using principal components analysis. The analysis controlled also for gender of the child, number of stool samples collected and number of anthelmintic treatments received. Maternal infection intensities with *A. lumbricoides* and *T. trichiura* were not included. Paternal STH infection was excluded from the multivariate model because of missing data.

## Discussion

In the present analysis, we investigated the epidemiology of and risk factors for STH infection during the first 3 years of life in a birth cohort from a largely rural District in tropical Ecuador. Over 40% of children had at least one STH infection documented during the first 3 years of life. Almost all infections (96.9%) were caused by *A. lumbricoides* and *T. trichiura*, although few of these pre-school children harboured heavy parasite burdens. Markers of poverty were independent risk factors for any STH infections or infections with individual parasites. STH infection risk in the cohort children was strongly associated with maternal STH infections during pregnancy, particularly children with mothers with moderate to high infection intensities with *A. lumbricoides* who had an approximately 12-fold increased risk of infection.

Potential limitations to the present study include losses to follow-up – we collected a stool sample from 70% of the original cohort at 3 years of age. Such losses could lead to selection bias. However, baseline variables were generally similar between those included and excluded from the analysis indicating that selection bias is probably not an important issue. Although we attempted to control for potential confounders, we cannot exclude confounding by uncontrolled factors or by highly correlated exposures as an alternative explanation for our findings. Approximately 76% of children were reported by mothers to have received at least one anthelmintic treatment during the first 3 years of life. This proportion did not vary significantly between infected and uninfected children indicating that mothers of children who did not receive anthelmintic treatment for their child for a positive stool examination were extremely likely to obtain anthelmintic drugs through other sources irrespective of a negative stool examination and concurs with our own experience that a lot of illness in children is attributed by mothers to the presence of ‘parasites’ and that self-medication is extremely common. Although 30% of all children had received 2 or more doses of anthelmintic drugs, number of treatments was not associated with risk of STH infections, an observation that might be explained by misclassification of this variable (number of anthelmintic treatments) or by high rates of reinfection over the year following treatment. The use of questionnaires to collect data on exposures is subject to reporting biases although these are unlikely to be systematic. Because data on risk factors was collected around the time of birth and before the measurement of outcomes, observation biases would seem unlikely to be important. Our findings are likely to be relevant to young children living in poor rural Districts of tropical Latin America and other similar regions elsewhere. Strengths of the study are the longitudinal nature of the study allowing repeated sampling of the same children over time and a large sample size. The study was originally designed and powered to examine the effects of maternal and early childhood infections with STH parasites on the development of atopy and allergic diseases [10]. These estimates that allowed losses to follow-up of 25% at 3 years for less common outcomes than childhood STH infections, nevertheless, had high power.

Most STH infections are unable to replicate within the human host and the acquisition of increasing parasite burdens is time and exposure-dependent. Unsurprisingly STH infection prevalence increased with age in the cohort with the highest prevalence of 24.9% observed at 36 months. This is within the 19.6–35.5% estimate by PAHO-WHO of STH prevalence in Ecuadorian pre-school children [14]. Age-specific estimates of prevalence and intensity may have been lower than expected in the present study because of the ethical requirement to provide treatment whenever a positive sample was detected. Schoolchildren would be expected to have a higher prevalence of STH infections: a previous study of schoolchildren living in two other rural Districts in Esmeraldas Province estimated a prevalence of 74.9% using the same diagnostic methods and a single stool sample [15]. The use of several diagnostic methods as done in the present study is useful to maximise the sensitivity for STH detection [16]. However, the methods we used have limited sensitivity for the detection of *S. stercoralis* and could be improved by using more sensitive molecular diagnostics such as PCR.

The strongest and most consistent risk factor for infection with STH in the first 3 years of life was maternal STH infections, particularly among children whose mothers harboured moderate to high parasite burdens with *A. lumbricoides* during pregnancy. A previous case-control analysis of a single stool sample collected from 1,004 children aged 7 months to 3 years, nested within the same cohort, showed that children of mothers infected with STH parasites during pregnancy had a higher risk of infection compared to children of uninfected mothers [9]. We now have extended these analyses to look prospectively at the acquisition of infection during the first 3 years of life in the whole cohort. Our data suggest that maternal STH infections, particularly moderate to heavy parasite burdens with *A. lumbricoides*, are an important independent determinant of risk of STH infections during early childhood.

The association between STH infections in mothers and infections of children has two possible explanations. 1) STH infections of the mother during pregnancy may increase susceptibility to infection in offspring through tolerization to parasite antigens - *A. lumbricoides* antigens can be detected in the circulation of infected individuals [17] that may cross the placenta and engage with the foetus's developing immune system. There is evidence from animal models of helminth infection [18] and studies in humans [19], [20], [21] that maternal infections may induce foetal tolerance to parasites and increase susceptibility to helminth infection in offspring. We have shown previously in the same study population that maternal STH infections induce immunologic sensitization to *A. lumbricoides* antigens *in utero* [22] and that the cord blood of newborns born to infected mothers has elevated levels of the immune regulatory cytokine IL-10 compared to those born of uninfected mothers [9]. Such tolerization could have a genetic basis, and there is ample evidence that susceptibility to STH infections is associated with immune genes [23], [24]. A recent study of children in urban Brazil provided evidence that IL-10 polymorphisms were associated with susceptibility to STH infections [25]. 2) The higher risk of infection in children of infected mothers may reflect a shared environment particularly during the first year of life when the child is completely dependent on the mother. This explanation is supported partly by the observation that among children whose primary carer during the first year of life was the mother, the association between maternal STH infection in pregnancy and any STH infection in offspring was stronger than for children whose primary carer was not the mother, although small numbers in the latter group yielded a very imprecise estimate.

Maternal STH infections were a common risk factor - approximately 46% of mothers were infected with STH in their third trimester of pregnancy, with an estimated attributable fraction of 27.9%. Our observations, therefore, have identified a potentially modifiable exposure - maternal STH infections - that could be evaluated in an intervention programme using currently available and highly efficacious anthelmintic treatments. An intervention in which anthelmintic drugs are given before pregnancy for women planning to have a family, during pregnancy or soon after birth or even periodically to women of child-bearing age could substantially reduce the risk of infection and potential morbidity during early childhood. Development of immune tolerance begins after 14 weeks gestation [26] so it might be beneficial to deworm women before pregnancy or if pregnant, in the second trimester before the foetal immune system is capable of developing tolerance to parasite antigens. Clearly, deworming of mothers before pregnancy would be preferable because giving anthelmintic drugs to pregnant or lactating women carries a risk of potential adverse effects on the foetus or neonate, respectively. Albendazole has been indicated by WHO for use in pregnant women after the first trimester in areas that are highly endemic for hookworm because of the risks to mother and child of maternal hookworm anaemia [27]. We now suggest another potential benefit of deworming mothers with other STH infections – to reduce the risk of STH infection and associated morbidity in early childhood. There are still limited data on the safety of anthelmintic drugs in pregnant and lactating women although benzimidazole drugs are believed to be safe when used after the first trimester or during breastfeeding [27]. Such a strategy (to reduce the risk of STH infection and associated morbidity in early childhood) will only be useful in areas where *A. lumbricoides* and *T. trichiura* are endemic because hookworm is less a problem in pre-school children among whom prevalence is generally low even in highly endemic areas [28]. The use of anthelmintic treatment in mothers, therefore, to prevent child infections will require careful consideration of the balance of potential adverse effects in the mother and child versus the clinical consequences of infections in the pre-school child for which there is growing evidence of important nutritional effects [29]. Such decisions will almost certainly have to be made locally depending on the epidemiology of STH parasites in any specific area or region. An alternative strategy would be the periodic treatment of women of childbearing age.

**Figure 4:**
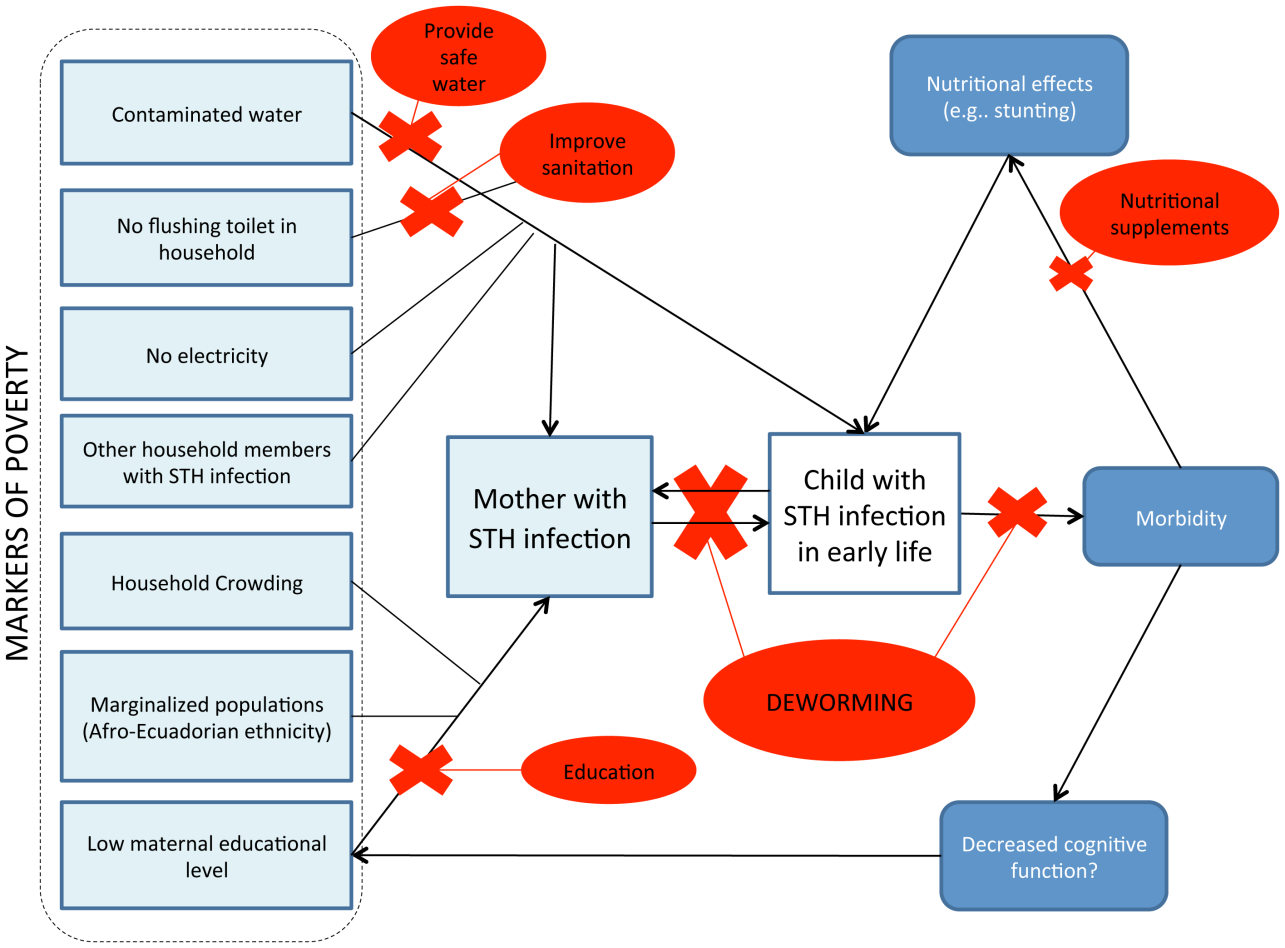
Model of the potential effects of risk factors on the risk of STH infections. The model shows potential effects of environmental and socioeconomic risk factors on risk of STH infections in early childhood and morbidity. Potential interventions to reduce risk of infection are illustrated with red crosses.

A model illustrating risk factors for childhood STH infections in our study with potential interventions is provided in Figure 4. Risk factors, in addition to maternal STH infections, identified in our analysis were markers of low socioeconomic status such as use of untreated water for drinking, and not having a flushing toilet in the house. Having a younger mother of Afro-Ecuadorian ethnicity, being lower in the birth order, and household overcrowding were also independently associated with childhood STH infections. Previous studies have identified similar factors as being associated with risk of STH infection [4], [17], [30], [31], [32], [33], [34]. Among public health interventions that could be implemented to reduce STH infection risk in pre-school children are the provision of sanitation, clean water, and education of mothers in appropriate hygienic behaviours. Significant changes in hygiene practices will be required to sustain any reductions achieved by chemotherapy [35]. However, treatment of mothers (ideally before pregnancy) and their young children or the periodic treatment of women of child-bearing age are the only strategies that can be implemented in the short-term with immediate benefits and without major municipal investment in local infrastructure and services. The targeting of anthelmintic treatment to pregnant women attending antenatal clinics, and mothers and their young children attending public vaccination clinics that serve the most vulnerable sections of the population in STH endemic areas, provides a rapid and easily implemented strategy for the control of STH infections in pre-school children.

In conclusion, our study identified risk factors for STH infection during the first 3 years of life in a birth cohort conducted in a rural District in coastal Ecuador. Over 40% of children were infected at least once with STH parasites during the first 3 years of life and risk factors for infections were those associated with poverty. We identified maternal STH infections as an important and potentially modifiable risk factor that could be evaluated in future intervention studies for the control of STH infections in pre-school children.

## Supporting Information

Checklist S1 STROBE Checklist.
(DOC)

Table S1 Comparison of covariables between children included (N = 1,697) and excluded (N = 707) from the analysis of the 2,404 newborns initially recruited.
(DOCX)

Table S2 Risk factors for any infection with *A. lumbricoides* or *T. trichiura* during the first 3 years of life.
(DOCX)

## References

[1] WHO (2012) Eliminating Soil-Transmitted Helminthiases as a Public Health Problem in Children. Geneva: World Health Organization.

[2] Hotez PJ, Bund DA, Beegle K, Brooker S, Drake L, et al.. (2006) Helminth Infections: Soil-Transmitted Helminth Infections and Schistosomiasis. Disease Control Priorities in Developing Countries. 2 ed. Washington: World Bank.

[3] Roy E, Hasan K, Haque R, Haque A, Siddique A, et al (2011) Patterns and risk factors for helminthiasis in rural children aged under 2 in Bangladesh. South African Journal of Child Health 5 3: 78–84.

[4] Wang X, Zhang L, Luo R, Wang G, Chen Y, et al (2012) Soil-Transmitted Helminth Infections and Correlated Risk Factors in Preschool and School-Aged Children in Rural Southwest China. PLoS ONE 7 9: doi:10.1371/journal.-pone.0045939 Available: http://www.plosone.org/article/info%3Adoi%2F10.1371%2Fjournal.pone.0045939 Accessed 11/01/13 10.1371/journal.pone.0045939 PMC3459941 23029330

[5] Oberhalman R, Guerrero E, Fernandez M, Silio M, Mercado D, et al (1998) Correlations between intestinal parasitosis, physical growth, and psychomotor development among infants and children from rural Nicaragua. American Journal of Tropical Medicine and Hygiene 58 4: 470–475. 9574794 10.4269/ajtmh.1998.58.470

[6] Hotez P, Brindley P, Bethony J, King C, Pearce E, et al (2008) Helminth infections: the great neglected tropical diseases. The Journal of Clinical Investigation 118 4: 1311–1321. 18382743 10.1172/JCI34261 PMC2276811

[7] De Silva N (2003) Impact of mass chemotherapy on the morbidity due to soil-transmitted nematodes. Acta Tropica 86: 197–214. 12745137 10.1016/s0001-706x(03)00035-4

[8] WHO (2012) Soil-transmitted helminthiases: number of children treated in 2010. Geneva. 24340403

[9] Mehta R, Rodriguez A, Chico M, Guadalupe I, Broncano N, et al (2012) Maternal Geohelminth Infections Are Associated with an Increased Susceptibility to Geohelminth Infection in Children: A Case-Control Study. PLoS Neglected Tropical Diseases 6 7: doi:10.1371/journal.pntd.0001753 Available: http://www.plosntds.org/article/info%3Adoi%2F10.1371%2Fjournal.pntd.0001753 Accessed 07/01/13. 10.1371/journal.pntd.0001753 PMC3404107 22848773

[10] Cooper P, Chico M, Guadalupe I, Sandoval C, Mitre E, et al (2011) Impact of early life exposures to geohelminth infections on the development of vaccine immunity, allergic sensitization, and allergic inflammatory diseases in children living in tropical Ecuador: the ECUAVIDA birth cohort study. BMC Infectious Diseases 11 184: doi:10.1186/1471-2334-11-184 Available: http://www.biomedcentral.com/1471-2334/11/184/ Accessed 05/07/12. 10.1186/1471-2334-11-184 PMC3141416 21714922

[11] Montresor A, Crompton D, Hall A, Bundy D, Savioli L (1998) Guidelines for the evaluation of Soil-Transmitted Helminthiasis and Schistosomiasis at community level. Geneva.

[12] Calvopiña M (1997) Terapéutica antiparasitaria. Ministerio de Salud Pública del Ecuador, 2^nd^ Edition. Ecuador.

[13] Ministerio de Salud Pública del Ecuador (2011) Atencion integrada a las enfermedades prevalentes de la Infancia: cuadros de procedimientos. Direccion de Promocion y Atencion Ecuador: Integral de Salud.

[14] Saboya M, Catala L, Ault S, Nicholls R (2011) Prevalence and intensity of infection of Soil-transmitted Helminths in Latin America and the Caribbean Countries: Mapping at second administrative level 2000–2010. Washington DC.

[15] Moncayo A, Vaca M, Oviedo G, Erazo S, Quinzo I, et al (2010) Risk factors for atopic and non–atopic asthma in a rural area of Ecuador. Thorax 65: 409–16. 20435862 10.1136/thx.2009.126490 PMC2988616

[16] Bukusuba J, Hughes P, Kizza M, Muhangi L, Muwanga M, et al (2004) Screening for intestinal helminth infection in a semi-urban cohort of pregnant women in Uganda. Tropical Doctor 34: 27–28. 14959970 10.1177/004947550403400113

[17] Tanaka K, Kawamura H, Tohgi N, Tsuji M, Miyachi Y, et al (1983) The measurement of *Ascaris suum* protein by radioimmunoassay in sera from patients with helminthiasis and with gastrointestinal disease. Parasitology 86: 291–300. 6682964 10.1017/s0031182000050459

[18] Rajan T, Bailis J, Yates J, Shultz L, Greiner D, et al (1994) Maternal influence on susceptibility of offspring to *Brugia malayi* infection in a murine model of filariasis. Acta Tropica 58 3–4: 283–289. 7709867 10.1016/0001-706x(94)90022-1

[19] Lammie P, Hitch W, Walker E, Hightower A, Eberhard M (1991) Maternal filarial infection as risk factor for infection in children. The Lancet 337 8748: 1005–1006. 10.1016/0140-6736(91)92661-k 1673168

[20] Steel C, Guinea A, McCarty J, Ottesen E (1994) Long-term effect of prenatal exposure to maternal microfilaraemia on immune responsiveness to filarial parasite antigens. The Lancet 343 8902: 890–893. 10.1016/s0140-6736(94)90009-4 7908359

[21] Malhotra I, Mungai P, Wamachi A, Tisch D, Kioko J, et al (2006) Prenatal T cell immunity to Wuchereria bancrofti and its effect on filarial immunity and infection susceptibility during childhood. Journal of Infectious Diseases 193: 1005–1013. 16518763 10.1086/500472

[22] Guadalupe I, Mitre E, Benitez S, Chico ME, Nutman TB, et al (2009) Evidence for *in utero* sensitization to *Ascaris lumbricoides* in newborns of mothers with Ascariasis. The Journal of Infectious Diseases 199: 1846–1850 doi:10.1086/599214 19426111 10.1086/599214 PMC2869024

[23] Williams-Blangero S, VandeBerg J, Subedi J, Jha B, Correa-Oliveira R, et al (2008) Localization of multiple quantitative trait loci influencing susceptibility to infection with *lumbricoides* . The Journal of Infectious Diseases 197: 66–71. 18171287 10.1086/524060

[24] Williams-Blangero S, VandeBerg J, Subedi J, Jha B, Dyer T, et al (2008) Two quantitative trait loci influence whipworm (*Trichuris trichiura*) infection in a Nepalese population. The Journal of Infectious Diseases 197: 1198–1203. 18462166 10.1086/533493 PMC4122289

[25] Figueiredo C, Barreto M, Alcantara-Neves N, Rodrigues L, Cooper P, et al (2013) Coassociations between IL10 polymorphisms, IL-10 production, helminth infection, and asthma/wheeze in an urban tropical population in Brazil. Journal of Allergy & Clinical Immunology 131 6: 1683–1690 doi:10.1016/j.jaci.2012.10.043 23273955 10.1016/j.jaci.2012.10.043 PMC5017514

[26] Cupedo T, Nagasawa M, Weijer K, Blom B, Spits H (2005) Development and activation of regulatory T cells in the human fetus. European Journal of Immunology 35: 383–390 doi:10.1002/eji.200425763 15682453 10.1002/eji.200425763

[27] WHO (2011) Assuring the safety of preventive chemotherapy interventions for the control of neglected tropical diseases. Geneva.

[28] Amberbir A, Medhin G, Alem A, Britton J, Davey G, et al (2011) The role of acetaminophen and geohelminth infection on the incidence of wheeze and eczema: a longitudinal birth-cohort study. American Journal of Respiratory and Critical Care Medicine 183 2: 165–170. 20935107 10.1164/rccm.201006-0989OC PMC3040388

[29] Albonico M, Allen H, Chitsulo L, Engels D, Gabrielli A-F, et al (2008) Controlling Soil-Transmitted Helminthiasis in Pre-School-Age Children through Preventive Chemotherapy. PLoS Neglected Tropical Diseases 2 doi:10.1371/journal.pntd.0000126. Available: http://www.plosntds.org/article/info%3Adoi%2F10.1371%2Fjournal.pntd.0000126 Accessed 15/01/13. 10.1371/journal.pntd.0000126 PMC2274864 18365031

[30] Muniz P, Ferreira M, Ferreira C, Conde W, Monteiro C (2002) Intestinal parasitic infections in young children in Sao Paulo, Brazil: prevalences, temporal trends and associations with physical growth. Annals of Tropical Medicine & Parasitology 96 5: 503–512. 12194711 10.1179/000349802125001311

[31] Belyhun Y, Medhin G, Amberbir A, Erko B, Hanlon C, et al (2010) Prevalence and risk factors for soil-transmitted helminth infection in mothers and their infants in Butajira, Ethiopia: a population based study. BMC Public Health 10 21: doi:10.1186/1471-2458-10-21 Available: http://www.biomedcentral.com/1471-2458/10/21 Accessed 07/01/13. 10.1186/1471-2458-10-21 PMC2835680 20085635

[32] Kounnavong S, Vonglokham M, Houamboun K, Odermatt P, Boupha B (2011) Soil-transmitted helminth infections and risk factors in preschool children in southern rural Lao People's Democratic Republic. Transactions of the Royal Society of Tropical Medicine and Hygiene 105: 160–166. 21288547 10.1016/j.trstmh.2010.11.011

[33] Cooper P, Chico M, Ordonez M, Gaus D, Griffin G (2003) Relationship between BCG vaccination, tuberculin sensitization, and infections with geohelminth infections. Transactions of the Royal Society of Tropical Medicine and Hygiene 97: 473–476. 15259485 10.1016/s0035-9203(03)90094-0

[34] Sackey M-E, Weigel M, Armijos R (2003) Predictors and Nutritional Consequences of Intestinal Parasitic Infections in Rural Ecuadorian Children. Journal of Tropical Pediatrics 49: 17–23. 12630715 10.1093/tropej/49.1.17

[35] Bieri F, Gray D, William G, Raso G, Li Y-S, et al (2013) Health-Education Package to Prevent Worm Infections in Chinese Schoolchildren. The New England Journal of Medicine 368 17: 1603–1612. 23614586 10.1056/NEJMoa1204885

