# Supplementary material for: Risk Factors for Soil-Transmitted Helminth Infections during the First 3 Years of Life in the Tropics; Findings from a Birth Cohort
Source: PLoS Negl Trop Dis. 2014 Feb 27;8(2):e2718. doi: 10.1371/journal.pntd.0002718 (PMC3937274; doi:10.1371/journal.pntd.0002718)
Supplement: Table S1 — Comparison of covariables between children included (N = 1,697) and excluded (N = 707) from the analysis of the 2,404 newborns initially recruited. (DOCX) [file pntd.0002718.s002.docx]

| Characteristic | Included  (n = 1697) | | Excluded  (n = 707) | | P value |
| --- | --- | --- | --- | --- | --- |
| Child factors |  |  |  |  |  |
| Sex (N, %) |  |  |  |  |  |
| Male | 865 | 51.0% | 363 | 51.3% |  |
| Female | 832 | 49.0% | 344 | 48.7% | 0.868 |
| Gestational age (weeks) (Mean, SD) | 39 | 2 | 39 | 2 | 0.75 |
| Birth order (N, %) |  |  |  |  |  |
| 1^st^ -2^nd^ | 823 | 48.5% | 372 | 52.6% |  |
| 3^rd^ -4^th^ | 546 | 32.2% | 200 | 28.3% |  |
| >5^th^ | 328 | 19.3% | 135 | 19.1% | 0.126 |
| Maternal factors | | | | | |
| Age (Mean, SD) | 26 | 6 | 25 | 6 | <0.001 |
| Ethnicity (N, %) |  |  |  |  |  |
| Afro-Ecuadorian | 446 | 26.3% | 170 | 24.0% |  |
| Other | 1251 | 73.8% | 537 | 76.0% | 0.408 |
| Educational level (N, %) |  |  |  |  |  |
| Illiterate | 258 | 15.2% | 110 | 15.6% |  |
| Complete primary | 1010 | 59.5% | 402 | 56.9% |  |
| Complete secondary | 429 | 25.3% | 195 | 27.6% | 0.436 |
| Paternal factors | | | | | |
| Age (Mean, SD) | 30 | 8 | 29 | 8 | 0.033 |
| Ethnicity |  |  |  |  |  |
| Afro-Ecuadorian | 379 | 23.0% | 162 | 23.8% |  |
| Other | 1270 | 77.0% | 519 | 76.2% | 0.253 |
| Educational level |  |  |  |  |  |
| Illiterate | 247 | 15.9% | 94 | 14.6% |  |
| Complete primary | 840 | 53.9% | 330 | 51.3% |  |
| Complete secondary | 470 | 30.2% | 219 | 34.1% | 0.199 |
| Socioeconomic factors | | | | | |
| Monthly income (US$) |  |  |  |  |  |
| 0-150 | 539 | 35.9% | 207 | 32.7% |  |
| 151-250 | 484 | 32.2% | 214 | 33.8% |  |
| ≥250 | 478 | 31.8% | 212 | 33.5% | 0.365 |
| Material goods |  |  |  |  |  |
| 0 | 126 | 7.4% | 59 | 8.3% |  |
| 1-2 | 696 | 41.0% | 318 | 45.0% |  |
| 3-4 | 875 | 51.6% | 330 | 46.7% | 0.091 |
| Electricity |  |  |  |  |  |
| No | 63 | 3.7% | 34 | 4.8% |  |
| Yes | 1633 | 96.3% | 673 | 95.2% | 0.214 |
| Environment | | | | | |
| Area of residence |  |  |  |  |  |
| Urban | 1180 | 69.5% | 505 | 71.4% |  |
| Rural | 517 | 30.5% | 202 | 28.6% | 0.355 |
| House construction |  |  |  |  |  |
| Wood/bamboo | 455 | 26.8% | 185 | 26.2% |  |
| Concrete-containing | 1242 | 73.2% | 522 | 73.8% | 0.744 |
| Bathroom |  |  |  |  |  |
| Field | 41 | 2.4% | 15 | 2.1% |  |
| Latrine | 1155 | 68.1% | 476 | 67.3% |  |
| WC | 501 | 29.5% | 216 | 30.6% | 0.818 |
| Drinking water |  |  |  |  |  |
| Potable |  |  |  |  |  |
| No | 1124 | 66.2% | 455 | 64.4% |  |
| Yes | 573 | 33.8% | 252 | 35.6% | 0.377 |
| Piped (untreated) |  |  |  |  |  |
| No | 1469 | 86.6% | 627 | 88.7% |  |
| Yes | 228 | 13.4% | 80 | 11.3% | 0.156 |
| Well |  |  |  |  |  |
| No | 845 | 49.8% | 351 | 49.6% |  |
| Yes | 852 | 50.2% | 356 | 50.4% | 0.948 |
| Household crowding |  |  |  |  |  |
| <3 people | 696 | 41.0% | 292 | 41.3% |  |
| ≥3 people | 1001 | 59.0% | 415 | 58.7% | 0.896 |
| Number of household members |  |  |  |  |  |
| 1-5 | 831 | 49.0% | 350 | 49.6% |  |
| 6-7 | 465 | 27.4% | 183 | 25.9% |  |
| ≥8 | 401 | 23.6% | 173 | 24.5% | 0.740 |
| Familial STH infections | | | | | |
| Mother |  |  |  |  |  |
| Any STH infection |  |  |  |  |  |
| No | 917 | 54.3% | 375 | 53.4% |  |
| Yes | 771 | 45.7% | 327 | 46.6% | 0.686 |
| *Ascaris lumbricoides* infection |  |  |  |  |  |
| No | 1231 | 72.9% | 491 | 69.9% |  |
| Yes | 457 | 27.1% | 211 | 30.1% | 0.139 |
| *A. lumbricoides* intensity |  |  |  |  |  |
| Negative | 1218 | 72.7% | 483 | 69.6% |  |
| 35-175 | 149 | 8.9% | 75 | 10.8% |  |
| 176-2770 | 155 | 9.3% | 67 | 9.7% |  |
| ≥2771 | 153 | 9.1% | 69 | 9.9% | 0.394 |
| *Trichuris trichiura* infection |  |  |  |  |  |
| No | 1212 | 71.8% | 491 | 69.9% |  |
| Yes | 476 | 28.2% | 211 | 30.1% | 0.361 |
| *T. trichiura* intensity |  |  |  |  |  |
| Negative | 1199 | 71.6% | 484 | 69.6% |  |
| 35-88 | 169 | 10.1% | 77 | 11.1% |  |
| 89-350 | 158 | 9.4% | 63 | 9.1% |  |
| ≥351 | 149 | 8.9% | 71 | 10.2% | 0.633 |
| Hookworm |  |  |  |  |  |
| No | 1593 | 93.9% | 672 | 95.0% |  |
| Yes | 104 | 6.1% | 35 | 5.0% | 0.260 |
| *Strongyloides stercoralis* |  |  |  |  |  |
| No | 1623 | 96.1% | 671 | 95.6% |  |
| Yes | 65 | 3.9% | 31 | 4.4% | 0.522 |
| Father |  |  |  |  |  |
| No | 1520 | 89.6% | 611 | 86.4% |  |
| Yes | 177 | 10.4% | 96 | 13.6% | 0.027 |
| Other household member |  |  |  |  |  |
| No | 899 | 53.0% | 330 | 46.7% |  |
| Yes | 798 | 47.0% | 377 | 53.3% | 0.005 |
| Number of stool samples |  |  |  |  |  |
| 0-1 | 158 | 9.3% | 55 | 7.8% |  |
| 2-4 | 781 | 46.0% | 345 | 48.8% |  |
| ≥5 | 758 | 44.7% | 307 | 43.4% | 0.314 |

Table S1. Comparison of covariables between children included (N=1,697) and excluded (N=707) from the analysis of the 2,404 newborns initially recruited.
