# Supplementary material for: Risk Factors for Soil-Transmitted Helminth Infections during the First 3 Years of Life in the Tropics; Findings from a Birth Cohort
Source: PLoS Negl Trop Dis. 2014 Feb 27;8(2):e2718. doi: 10.1371/journal.pntd.0002718 (PMC3937274; doi:10.1371/journal.pntd.0002718)
Supplement: Table S2 — Risk factors for any infection with A. lumbricoides or T. trichiura during the first 3 years of life. (DOCX) [file pntd.0002718.s003.docx]

Table S2. Risk factors for any infection with *A. lumbricoides* or *T. trichiura* during the first 3 years of life.

| Variable | Any A. lumbricoides infection | | Any T. trichura infection | |
| --- | --- | --- | --- | --- |
|  | OR (95% CI) | P value | OR (95% CI) | P value |
| Child Factors | | | | |
| Birth Order: <5^th^ vs. ≥5^th^ | 1.39 (0.97-1.99) | 0.078 | 1.42 (1.01-2.0) | 0.042 |
| Maternal Factors | | | | |
| Age: <26 vs. ≥26 years | 1.41 (1.05-1.89) | 0.021 |  |  |
| Ethnicity: Afro vs. Other | 1.72 (1.31-2.25) | <0.001 | 1.88 (1.40-2.55) | <0.001 |
| Educational level |  |  |  |  |
| Primary vs. Illiterate | 0.62 (0.44-0.86) | 0.005 | 0.73 (0.50-1.06) | 0.094 |
| Secondary vs. Illiterate | 0.38 (0.24-0.58) | <0.001 | 0.51 (0.31-0.84) | 0.008 |
| Socioeconomic Factors | | | | |
| SES Index |  |  |  |  |
| Medium vs Low | 0.77 (0.57-1.03) | 0.082 |  |  |
| High vs Low | 0.64 (0.47-0.89) | 0.007 |  |  |
| Environmental Factors | | | | |
| Area of residence: Urban vs. Rural | 1.38 (1.04-1.83) | 0.027 | 1.64 (1.18-2.28) | 0.003 |
| Household crowding: ≥3 vs. <3 | 1.43 (1.07-1.93) | 0.017 | 1.69 (1.17-2.43) | 0.005 |
| Maternal STH Infections | | | | |
| *A. lumbricoides* intensity (epg) | |  |  |  |
| Light vs. Negative | 1.18 (0.89-1.58) | 0.256 | 1.87 (1.36-2.56) | <0.001 |
| Moderate/high vs. Negative | 3.88 (2.12-7.08) | <0.001 | 5.85 (3.29-10.4) | <0.001 |
| *T. trichiura:* Yes vs. No | 1.38 (1.05-1.82) | 0.021 | 1.71 (1.36-2.56) | <0.001 |
| Hookworm: Yes vs. No |  |  |  |  |
| Household member with STH infection: Yes vs. No | 1.32 (1.02-1.71) | 0.035 | 1.39 (1.02-1.88) | 0.035 |

ORs and 95% CIs were estimated using multivariable logistic regression. Analyses controlled also for number of stool samples and anthelmintic treatments received. Socioeconomic status represents tertiles of z scores obtained using a factor analysis. Crowding is defined as the number of people living in the household per sleeping room. STH infections were detected using direct saline, modified Kato-Katz and formol-ether concentration methods. Infection intensities were estimated using the Kato-Katz method. STH infection intensity categories were: *A. lumbricoides* (light- <5,000 eggs per gramme of stool [epg];; moderate = 5,000-49,999; heavy – ≥50,000); *T. trichiura* (light - <1,000 epg; moderate – 1,000-9,999; heavy – ≥10,000).
